# Personalized repetitive transcranial magnetic stimulation (prtms®) for post-traumatic stress disorder (ptsd) in military combat veterans

**DOI:** 10.1016/j.heliyon.2023.e18943

**Published:** 2023-08-08

**Authors:** Milan T. Makale, Shaghayegh Abbasi, Chad Nybo, Jason Keifer, Lori Christman, J. Kaci Fairchild, Jerome Yesavage, Kenneth Blum, Mark S. Gold, David Baron, Jean Lud Cadet, Igor Elman, Catherine A. Dennen, Kevin T. Murphy

**Affiliations:** a Department of Radiation Medicine and Applied Sciences, University of California San Diego, La Jolla, CA, 92093, USA; b Department of Electrical Engineering, University of Portland, Portland, OR, 97203, USA; c CrossTx Inc., Bozeman, MT, 59715, USA; d Brain Health Hawaii, Honolulu, HI, 96816, USA; e StatKing Clinical Services, Fairfield, OH, 45014, USA; f Psychiatry and Behavioral Sciences, Stanford University, Stanford, CA, 94305, USA; g Sierra Pacific Mental Illness Research, Education, and Clinical Center, VA Medical Center, Palo Alto, CA, 94304, USA; h Division of Addiction Research & Education, Center for Sports, Exercise & Global Mental Health, Western University Health Sciences, Pomona, USA; i Department of Clinical Psychology and Addiction, Institute of Psychology, Faculty of Education and Psychology, Eötvös Loránd University, Hungary; j Department of Psychiatry, Wright University, Boonshoft School of Medicine, Dayton, OH, USA; k Department of Molecular Biology and Adelson School of Medicine, Ariel University, Ariel, Israel; l Department of Psychiatry, Washington University School of Medicine, St. Louis, MO, USA; m Molecular Neuropsychiatry Research Branch, National Institute on Drug Abuse, Intramural Research Program, National Institutes of Health, Baltimore, MD, USA; n Cambridge Health Alliance, Harvard Medical School, Cambridge, MA, USA; o Department of Family Medicine, Jefferson Health Northeast, Philadelphia, PA, USA; p PeakLogic Inc., Del Mar, CA, 92130, USA

**Keywords:** Alpha oscillations, Automatic cognition, Contextual, Default mode network, EEG, Experience-based, Fast thinking, Implicit, Intuitive, Power spectrum, PTSD, PTSD checklist for DSM-5 (PCL-5), rTMS

## Abstract

Emerging data suggest that post-traumatic stress disorder (PTSD) arises from disrupted brain default mode network (DMN) activity manifested by dysregulated encephalogram (EEG) alpha oscillations. Hence, we pursued the treatment of combat veterans with PTSD (n = 185) using an expanded form of repetitive transcranial magnetic stimulation (rTMS) termed personalized-rTMS (PrTMS). In this treatment methodology spectral EEG based guidance is used to iteratively optimize symptom resolution via (1) stimulation of multiple motor sensory and frontal cortical sites at reduced power, and (2) adjustments of cortical treatment loci and stimulus frequency during treatment progression based on a proprietary frequency algorithm (PeakLogic, Inc. San Diego) identifying stimulation frequency in the DMN elements of the alpha oscillatory band. Following 4 - 6 weeks of PrTMS® therapy in addition to routine PTSD therapy, veterans exhibited significant clinical improvement accompanied by increased cortical alpha center frequency and alpha oscillatory synchronization. Full resolution of PTSD symptoms was attained in over 50% of patients. These data support DMN involvement in PTSD pathophysiology and suggest a role in therapeutic outcomes. Prospective, sham controlled PrTMS® trials may be warranted to validate our clinical findings and to examine the contribution of DMN targeting for novel preventive, diagnostic, and therapeutic strategies tailored to the unique needs of individual patients with both combat and non-combat PTSD.

## 1 Introduction

Post-traumatic stress disorder (PTSD) is the fourth most common psychiatric disorder in adults, and it often seriously degrades cognitive functioning and emotional stability. About 30% of PTSD patients are still afflicted more than 10 years after diagnosis [1,2]. The situation with military combat veterans is especially difficult, with PTSD being highly prevalent and particularly treatment resistant [[1], [2], [3], [4], [5]]. According to the U.S. Veterans Affairs National Center for PTSD, 47% of patients undergoing trauma-based psychotherapy will fail to achieve remission, while medication alone results in a 58% treatment failure rate [6]. A recent (2021) literature review meta-analysis revealed that PTSD treatment dropout rates are high, up to 28.5% in civilians, and reach 38.5% in military active-duty personnel and veterans [[7], [8], [9]]. Escalated PTSD therapies include monoamine oxidase (MAO) inhibitors, which are limited by hazardous drug interactions, and electroconvulsive (ECT) therapy, which is an expensive hospital procedure, and can be risky in patients with cardiovascular conditions [5,[8], [9], [10], [11]]. Hence, novel PTSD therapies that are efficacious, well tolerated and cost effective are in high demand [1,5].

PTSD is characterized by reliving the event via intrusive memories, flashbacks and nightmares, avoidance reminders of the trauma and the resultant anxious symptomatology disrupting the lives of patients and of their loved ones [12]. To date, most psychophysiology [[13], [14], [15]] and functional brain imaging PTSD studies [16,17] have focused on conditioned fear [18,19] underlying enhanced- or unsuccessfully extinguished responses to trauma-related cues [20]. However, our recent research [21] also links PTSD with aberrations in contextual processing [22,23]. Contextual processing refers to the adaptive way the brain interprets incoming sensory information in the context of environment and prior experiences. As such, it involves the processing of multiple sources of information, including sensory input, memories, emotions, and salience, to generate a cohesive and meaningful representation of the current situation [24]. This activity enables instantaneous and spontaneous decision making in contrast to “slow thinking” [25] i.e., cost/benefit-informed deliberate and quantitative cognition aimed at profit maximization [21]. Individuals with PTSD often have difficulty properly integrating sensory information [[26], [27], [28], [29], [30], [31]], leading to autonomic instability, hyperarousal and hypervigilance in response to environmental stimuli [12]. By better understanding the neural mechanisms underlying these deficits, researchers may be able to develop more effective treatments for PTSD that target its underlying cognitive and neural abnormalities.

A key brain network involved in contextual processing is the default mode network (DMN), which includes the cingulate cortex, medial prefrontal cortex, cuneus/precuneus, and temporoparietal junction/angular gyrus, which become active when an individual is not engaged in a specific task or focused on the external world [[32], [33], [34]]. The DMN is involved in the integration of information from multiple brain regions and the generation of a coherent sense of self including autobiographical memory, self-referential thought, and social cognition.

Importantly, PTSD is associated with altered DMN function, including increased connectivity within the network and disrupted connectivity [23] between the DMN and other brain regions involved in cognitive control [[35], [36], [37], [38]]. Both may be implicated [39] in PTSD's “core component” of implicit memories [[40], [41], [42]] contained within the re-experiencing “B” diagnostic criteria along with the automatic [43] “negative alterations in cognitions” [44,45] that are encoded within in “D” diagnostic criteria e.g., negative thoughts and assumptions [12]. Furthermore, implicit trauma-related cues [46,47], and irrational [48,49] decision making may worsen PTSD symptoms [12] while psychotherapeutic and psychopharmacologic therapeutic approaches targeting automatic processing [[50], [51], [52]] namely, Eye Movement Desensitization and Reprocessing [53] or psychedelics [54] seem to exert beneficial effect for PTSD patients.

Since by its nature spontaneous and automatic cognition is not readily accessible to self-reports [21] inquiry into the DMN's role in the PTSD therapeutic outcomes is limited, in part, by a paucity of robust laboratory-based procedures that may measure real time treatment-related adjustments in DMN function. To that end we have developed [55] a repetitive transcranial magnetic stimulation (rTMS) protocol termed personalized-rTMS (PrTMS) which incorporates the key electroencephalographic measure of DMN activity [56], the alpha oscillatory rhythm (8–13 Hz). This guides the pursuit of optimal PTSD symptom resolution via: (a) stimulation of multiple motor sensory and frontal cortical sites at reduced power, and (b) iterative adjustments of cortical treatment loci and stimulus frequency during treatment progression. Stimulation sites and frequencies are identified via a proprietary frequency algorithm and computer code (Python; PeakLogic, Inc. San Diego) that quantitatively analyzes the spectral EEG and targets elements of the DMN relevant to the alpha oscillatory band [34].

If dysregulated alpha oscillation in the DMN creates clinical disturbances, it is reasonable to expect amelioration of these alterations via stabilization of alpha activity [57,58]. Hence, alpha band oscillatory frequency may be synchronized and reset via rTMS, which, as suggested in a recent meta-analysis, normalizes PTSD symptomatology [[59], [60], [61]]. However, the directionality of the PTSD symptomatology response is not entirely consistent [62,63] as rTMS has reportedly induced improvement [[64], [65], [66]], worsening [67] and no change [68]. This may be particularly the case for combat Veterans given the high rates of comorbidities [69,70] e.g., traumatic brain injury, chronic pain, major depression, anxiety disorders, and substance abuse disorders [69,[71], [72], [73], [74], [75]].

For instance, a sham-controlled study with 62 Veterans comparing 1 Hz rTMS to the right dorsolateral prefrontal cortex (R-DLPFC) plus cognitive processing therapy (CPT), versus sham rTMS plus CPT [76] reported 4- and 8-point reductions in mean PTSD Checklist for Diagnostic and Statistical Manual of Mental Disorders (PCL) scores, respectively, at 4-week and 6-month follow-ups, which were statistically significant compared to sham rTMS. In contrast, a multicenter Veterans Administration (VA) study taken to completion on 125 veterans, showed that active rTMS at 10 Hz to the left DLPFC (L-DLPFC) did not elicit a greater effect than did sham rTMS. The PTSD PCL-M score reductions were respectively, 5.2 versus 8.1-points, signifying a placebo effect [5,59].

PrTMS has not previously been attempted in combat-related PTSD. The aim of the present work was to examine the clinical outcome effects of PrTMS when added to ongoing therapy in Veterans with combat PTSD. Given the reported beneficial outcomes of standard rTMS in combat Veterans [63], we hypothesized that an added EEG guidance component and stimulation of multiple targeted cortical sites would lead to superior outcomes. These outcomes were predicted to include reduced PCL-5 scores along with Hamilton Anxiety Rating Scale (HAM-A)- and Hamilton Depression Rating Scale (HAM-D) scores, and with a corresponding shift in the spectral EEG alpha band peak center frequency to a lower level, and increased cortical alpha peak synchrony according to robust regression analysis of the spectral EEG.

## 2 Methods

### 2.1 Subjects

Clinical screening, assessment and treatment were done by qualified physicians and medical technicians in an active rTMS medical clinic (MindSet, Inc.) located in Del Mar, California. Male and female veterans (n = 300) that had served in a combat zone were initially screened for PTSD symptoms using the 33 cutoff PCL-5 score [77]; those with a pre-treatment baseline PCL-5 score <33 were excluded [78]. The age range was about 34–75 years with a mean of almost 53 years, and there was approximately a 1:1 ratio of males to females. We felt that formal diagnoses were not essential as the patients were all military combat veterans and a positive PCL-5 self-report questionnaire would very likely signal the presence of PTSD. Subsequent eligibility screening included stable medications and psychotherapeutic regimes for at least 8 weeks prior to the enrolment as well as rTMS safety and exclusion criteria [[79], [80], [81]]. Accordingly, subjects were excluded based on diagnoses of a major psychiatric illness other than PTSD such as bipolar disorder, schizophrenia spectrum disorder, and major depression. Potential patients were disqualified if they had a previous history of psychosis, were taking anti-psychotics, had mania, had bipolar disorder, and if they had ferrous metal in the head. Pain and substance use disorders were not disqualifying, as ours was a treatment program. All patients were briefed on PrTMS procedures and they provided informed medical consent to be treated. Patients continued their standard psychotherapy and/or medication(s) during the course of PrTMS treatment. Secondary psychometric outcomes were the Hamilton Anxiety Rating Scale (HAM-A) and Hamilton Depression Rating Scale (HAM-D) scores [82].

#### 2.1.1 Declarations

After the procedures were fully explained, all subjects gave written informed consent and the protocol was approved by an institutional review board (IRB): WCG IRB Study number 1254094; IRB tracking number 20190239. Patients continued their standard psychotherapy and/or medication(s) during the course of PrTMS treatment.

### 2.2 Therapeutic protocol

**Fig. 1:**
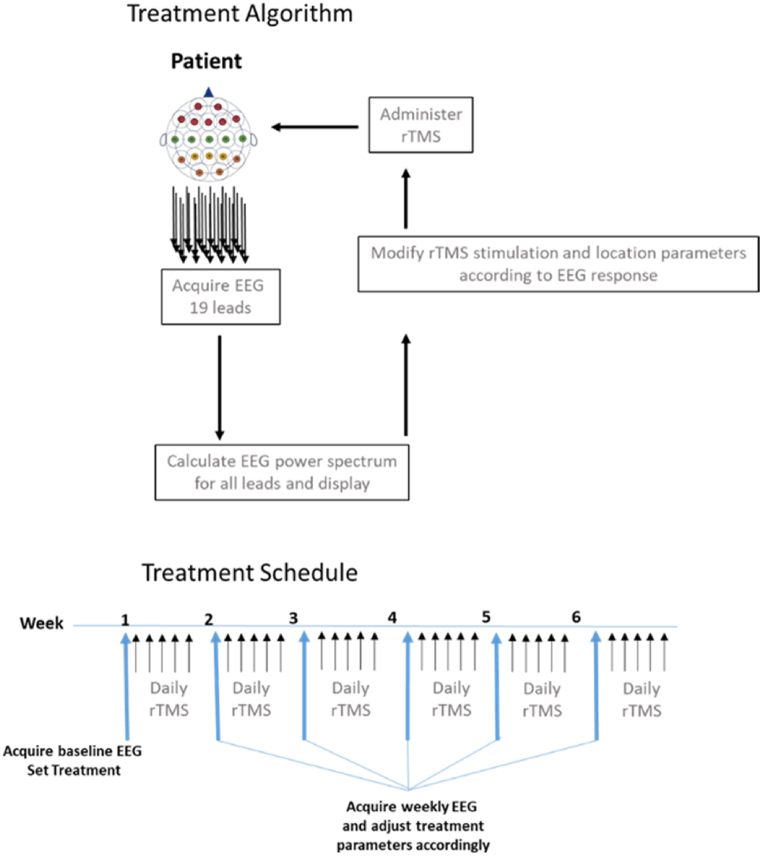
Patient PrTMS treatment algorithm and the treatment schedule. Patient EEG recordings were acquired once every week for 4–6 weeks, in some cases longer, and the power spectrum derived from the EEG was used to determine what stimulation frequency and what cortical locations were treated. Patients received 5 daily treatments each week.

The study protocol was aligned with the Strengthening the Reporting of Observational studies in Epidemiology (STROBE) checklist and guidelines [83]. A treatment session was applied daily, 5 days per week typically for 4–6 weeks with a range of 4–22, and one subject remained at 80 weeks. (Fig. 1). Clinical personnel evaluated patients daily for adverse events (AEs). These included headache, scalp pain, cognitive deficits, and seizures. AEs also included observed or self-reported problems, complaints, physical signs and symptoms, medical conditions occurring during treatment that were not previously present, and previous medical conditions that worsened. Adverse event severity was assessed according to the following criteria: mild awareness of discomfort but easily tolerated, moderate discomfort enough to cause interference with usual activity, severe incapacitating discomfort with the inability to perform work or usual activities.

Presently there is no consensus on what specific cortical site(s) and stimulation frequencies may be required for rTMS treatment of PTSD patients [62]. Our PrTMS approach, based on the clinical experience acquired by our lab over the course of years, incorporates rTMS guidance via electroencephalogram (EEG) spectral analysis. We deliver frequency specific stimulation of an extensive cortical area encompassing discrete motor, sensory, and prefrontal sites [84] while optimally reducing the TMS power without therapeutic loss [84,85] by adjusting the anatomical loci and stimulation frequency on a weekly basis as the therapy progresses.

At the onset of each treatment week patients completed PCL-5, HAM-A and HAM-D questionnaires and EEG. The questionnaire and EEG data guided the weekly adjustments of PrTMS stimulus' frequencies and cortical treatment sites. EEG recordings were acquired before PrTMS and at the beginning of each week as long as PrTMS continued, usually over approximately 6 weeks or up to 28 daily weekday treatment sessions, excluding weekends. The total number of sessions aligns with a previous report of rTMS in veterans with PTSD, and exceeds the average number of rTMS sessions, 16 (range:10–40), for 9 other PTSD studies [5,62]. The EEG was recorded from awake, eyes closed, seated subjects using a 19-lead high impedance dry electrode EEG headset (Cognionics [CGX] Inc., San Diego CA). Neuronavigation was not used, rather the locations were determined by the EEG data from each electrode arranged according to the standard 10–20 system. The power spectra from each electrode were analyzed, and those locations that exhibited an alpha center frequency that deviated from the subject's intrinsic alpha center frequency were stimulated at the intrinsic frequency. The intrinsic frequency was identified from the alpha center frequency of the occipital electrodes, since visual cortical frequency tends to be preserved, even with psychopathology.

Following stimulus frequency selection, treatment was delivered by a trained rTMS technician using a MagVenture MagPro R30 transcranial stimulator and B-65 head transducer. Patients were seated in a quiet room with their eyes closed and without sedation. The selected magnetic field intensity was comparatively reduced and was gradually increased over the course of treatment (Fig. 1). Stimulation intensity was 25–60% of the resting motor threshold in most patients, and the stimulus frequency range was 8–13 Hz, with magnetic pulses delivered in 10–15 s trains. Intertrain intervals began at 30 s, and gradually decreased to 10 s. During each treatment session, which lasted about 40 min, the motor-sensory strip and subsequent prefrontal and frontal regions were treated in succession. The EEG was recorded from awake, eyes closed, seated subjects using a 19-lead high impedance dry electrode EEG headset (Cognionics [CGX] Inc., San Diego CA). For spectral variables, the frontal cortical region included EEG leads FP1 to F8, cortical region 2 was central and contained leads Cz to T4, cortical region 3 was parietal and contained leads Pz to P4, and cortical region 4 was occipital and contained leads P7 to O2.

EEG data pre-processing included visual inspection and removal of distinctly erratic and technically flawed recordings identified by experienced technicians who were ‘blind’ to the study design and hypotheses. In line with established procedures, filtering, and selective removal of EEG recordings (if any) was avoided as much as possible [86]. A 4-min EEG time epoch was transformed via Welch's Fast Fourier Transform (FFT) employing a custom Python program, to produce a power spectrum with 0.1 Hz resolution; the spectral frequency band was restricted to between 2 and 20 Hz in the power spectrum. The extracted alpha band (8–13 Hz) power spectrum used in subsequent analyses is devoid of low frequency artifacts obviating the necessity of filtering with consequent potential for bias. A proprietary spectral EEG analysis algorithm (*PeakLogic, Inc*. San Diego) identified an initial stimulation frequency in the alpha band, and continually adjusted this frequency as a function of the change in objective alpha wave characteristics, according to successive EEG power spectral acquisitions, and clinical response, as measured by the psychometric questionnaires. The power spectral amplitude center frequency in the alpha band between 8 and 13 Hz was determined weekly for each PCL-5 PrTMS responder and each nonresponder (see Results section below), for each EEG electrode.

### 2.3 Data processing

#### 2.3.1 Symptom data

The primary PrTMS efficacy endpoint was the reduction in symptoms measured by the DSM-5 PCL-5 total score, acquired weekly from baseline (pretreatment) to week 4, week 6 and to final treatment. Treatment efficacy was defined as a statistically significant reduction in mean PCL-5 total score compared to baseline. Observed (raw) and change from baseline (CFB) PCL-5 scores were summarized in terms of the number of non-missing observations (n), mean, standard deviation (SD), median, and range by time point.

The following hypotheses on the mean CFB data were tested for the fourth- and last treatment time points using a *t*-test at a two-sided α = 0.05 level of significance: H_0_: μcfb = 0 vs. H_1_: μcfb ≠ 0. For PCL-5 changes the mean CFB data were tested using a *t*-test at a two-sided α = 0.05 level of significance: H_0_: μcfb = 0 vs. H_1_: μcfb ≠ 0. The null hypothesis would be rejected in favor of the alternative at the two-sided p-value <0.05.

The number of patients exceeding the change thresholds induced by PrTMS, as measured with the PCL-5, was compared retrospectively to the corresponding numbers to our prior active and sham rTMS study [66] employing DSM-IV PCL-Military (PCL-M). Notably, PCL-5 is based on the DSM-5 symptoms, and it is distinct from PCL-M. While both utilize the same Likert type rating scale descriptors, and exhibit continuity, the number of questions differs, 20 versus 17 respectively, and the rating scales for each question are different rendering the scales incompatible for interchangeable use [87]. Nonetheless, the VA Center for PTSD recommends, based on statistical validation, the same score change thresholds and ranges to assess the treatment progress. Specifically, 5 points change denotes a reliable treatment response, not due to a chance, whereas 10-point change is considered to be clinically meaningful [88] for both tools [89].

The HAM-A and HAM-D score changes with PrTMS were analyzed with a paired two-sided parametric *t*-test which compared the prior to treatment values to those acquired at 6-week timepoint. The null hypothesis was that the mean HAM-A/HAM-B pretreatment score was equal to the mean HAM-A/HAM-B score at six weeks, and the significance threshold was p < 0.05. For the HAM-A a score of 8–14 indicates mild anxiety, 15–23 indicates moderate anxiety, and greater than 24 indicates severe anxiety [90]. A HAM-D score of 0–7 is considered normal, 8–16 suggests mild depression, 17–23 is moderate depression, and scores over 24 indicate severe depression [91].

The dominant alpha peak (center) frequency was determined for all EEG leads, averaged for each cortical region, and a nonparametric binomial distribution sign test compared binned data before and after PrTMs. The amplitude of the alpha band (8–13 Hz) spectral center frequency was identified for each EEG lead up to 6 weeks of treatment, yielding 728,688 and 101,802 data points for responders and nonresponders, respectively. For each week, and for each patient the peak amplitudes for all the frontal electrodes, and also for the entire brain cortex, were averaged for all responders and nonresponders. The alpha band (8–13 Hz) center peak full width half max (FWHM) was analyzed with a custom Python program, utilizing *scipy.signal* and *scipy.stat* modules. The 1/f aperiodic spectral component was determined by averaging the 2–20 Hz power spectrum amplitude from the 7 leads in the frontal cortex, plotting log power versus log frequency, and then calculating the robust regression line which treated periodic oscillatory components as outliers [[92], [93], [94], [95]].

## 3 Results

### 3.1 Attrition

Out of the screened cohort, 195 subjects had PCL-5 scores of 33 or greater warranting the PTSD diagnosis [77]. All subjects tolerated the application of rTMS to multiple cortical sites well and reported only occasional mild and transient discomfort (if any), and no serious adverse events such as seizures, and there were no effects that needed treatment. This favorable tolerability profile may be attributable to relatively lower magnetic field strengths utilized in the present study. To ascertain a meaningful effect size, the *a priori* focus was placed on the 4 – 6-week period analyses, since by 27 treatments the dropout rate was substantial, 31%, 60 patients left the study, and at week 22 the dropout rate was 92%, 180 patients left the study, and only one patient remained at week 80.

### 3.2 Psychometrics

**Fig. 2:**
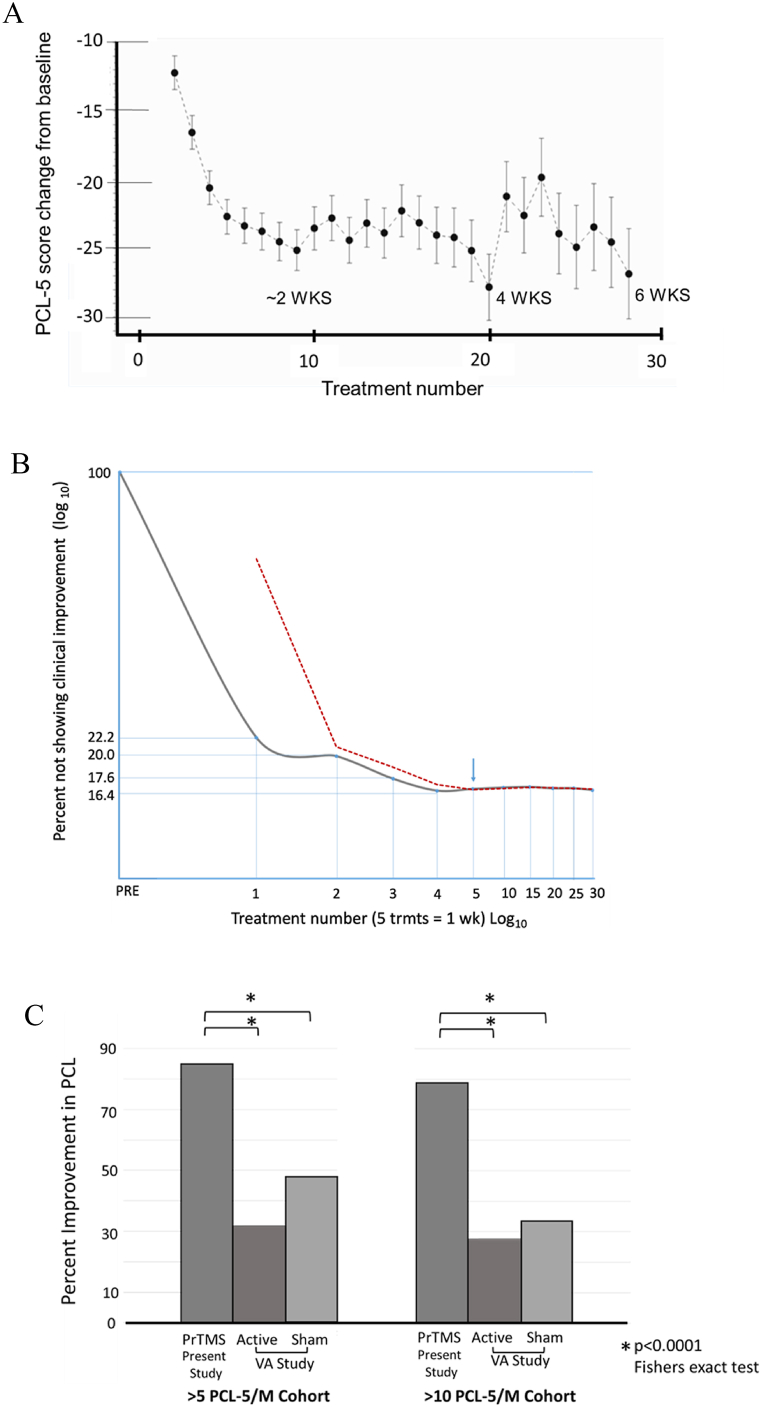
a) Change in PCL-5 scores with treatment number. The graph shows the least squares means and 95% confidence limits, according to treatment number. After 28 treatments patients dropped out sporadically so that too few patients remained per time point. Only treatment points with 10 patients or more are included. The graph indicates up to 27 treatments or almost about 6 weeks. b) Percentage of patients with no clinical improvement according to PCL-5 score, versus the number of PrTMS sessions (treatments). The log-log plot chronicles progress over 27 sessions or almost 6 weeks. The patients that showed score improvement did so over the first week, which typically equated to 5 successive treatments. The red dotted line denotes the moving average and its slope becomes horizontal at about 5 weeks (arrow), and no tangible improvement was seen after approximately 4 weeks. c) Proportion of patients exceeding PCL-5 change thresholds for PrTMS and for rTMS. Percentage of patients exceeding PCL-5 change thresholds (>5 pts and >10 pts) for PrTMS versus previous VA study data of active standard rTMS and Sham rTMS in depression and PTSD. Fishers exact test p < 0.0001 for all comparisons shown.

The mean baseline (pre-treatment) PCL-5 score was 53.3 ± SD = 11.46; median = 52 (Table 1, Table 2). Subjects showed significant reductions in PTSD symptomatology after 5 treatments, by the week 2 timepoint. The mean CFB at each treatment timepoint is depicted in Fig. 2a, plotted with least squares confidence limits. The mean CFB was statistically significant at the 4- and 6-week time points (p < 0.0001 each; Table 1, Table 2). Mean peak reduction in PCL-5 score was 20.6 points at the week 4 timepoint, to a mean score of 32.7 (p < 0.0001). Thus, by week 4 in over half of the subjects (n = 96) the PCL-5 score was below the PTSD diagnostic threshold (Table 1). Subjects continued to show improvement over time, and their improvement was almost entirely complete at week 4, albeit at a plateauing rate (Fig. 2a and b). As indicated in Table 2, the mean score for the entire treatment period beyond the 6-week timepoint was 28.8 ± 18.77, a mean reduction of 24.5 PCL-5 points.

**Table 1:** Change from baseline to fourth treatment week for patients with an initial PCL-5 score of 33 or higher. Maximum score reduction at 4 weeks for treatment responders and non-responders.

Weeks Treated	Data Type^a^	n	Mean	Std Dev	Median	Min	Max	*P*-Value^b^
1	RAW	195	53.3	11.46	52	33	80	
4	RAW	195	32.7	18.12	32	0	80	
4	CFB	195	−20.6	16.64	−18.0	−63	17	<0.0001
Week 4 PCL-5 <32	RAW	96	17.7	9.44	18.5	0	31	
Week 4 PCL-5 >32	RAW	99	47.2	11.40	44.0	32	80	<0.0001

a RAW = observed data. CFB = change from baseline = post-baseline score –baseline score.

b At Week 4, two-sided p-value for test of H0: Mean CFB = 0 vs. H1: Mean CFB not equal to 0.

**Table 2:** Change from baseline to final treatment for patients with a Pre-PrTMS PCL-5 score of 33 or higher.

Data Type^a^	n	Mean	Std Dev	Median	Min	Max	*P*-Value^b^
RAW	195	28.8	18.77	28.0	0	79	
CFB	195	−24.5	18.09	−24.0	−71	23	<0.0001
Week 4 Score <32	RAW	96	17.7	9.44	18.5	0	31
Week 4 Score >32	RAW	99	47.2	11.40	44.0	32	80

a RAW = observed data. CFB = change from baseline = post-baseline score –baseline score.

b At final, two-sided p-value for test of H0: Mean CFB = 0 vs. H1: Mean CFB not equal to 0.

**Fig. 3:**
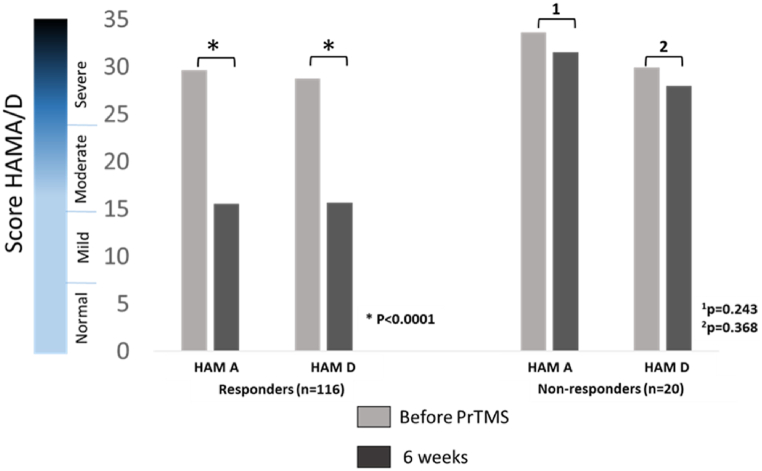
Comparison of self-reported HAM-A and HAM-D scores before and after PrTMS. This is depicted for subjects that either responded or did not respond to PrTMS in terms of PCL-5 score reduction of >5 points. The scale on the left indicates approximate severity level, and there is overlap between the HAM-A and Ham-D in this context. PrTMS in responders (n = 116) was associated with highly significant anxiety and score reductions (parametric paired *t*-test, p < 0.0001) while there was no significant change for nonresponders (n = 19, HAM A, p = 0.243, and HAM D, p = 0.368).

In the present project, responders (85%) and non-responders (15%) were defined based on the VA Center for PTSD criteria [89] i.e., respective presence or absence of a greater than a 5-point PCL-5 score drop after PrTMS (Table 3). Sustained improvement starting at Week 1 after the conclusion of the fifth treatment was reflected in the corresponding percentage of subjects’ improvement throughout the entire study (Fig. 2a). Fig. 2b shows that the percentage of patients exhibiting PCL-5 score improvement did not change appreciably after the 5th treatment, suggesting that the plateau after 5 weeks seen in Fig. 2a may represent those patients that had not responded to therapy. Fig. 3 shows HAM-A and HAM-D scores for the responders and non-responders. Given the heightened prevalence of anxiety and depressive symptomatology in PTSD patients [96], as expected, in responders the scores dropped to clinically negligible values whereas in non-responders these scores remained at the same heightened level throughout the entire study.

**Table 3:** PCL-5 data for PrTMS at 4 weeks of treatment, minimum score for responders and nonresponders.

Group	Data Type^a^	n	Mean	Std Dev	Median	Min	Max
Week 4 Score <32	RAW	96	17.7	9.44	18.5	0	31
Week 4 Score >32	RAW	99	47.2	11.40	44.0	32	80

### 3.3 PrTMS vs. standard rTMS in VA study: outcome of active and sham treatment

In our prior multi-center VA study with rTMS, depression, and PTSD in veterans, standard rTMS was used to stimulate one prefrontal cortical site using a common pulse frequency [5]. Active rTMS vs. sham rTMS [5] produced a greater than 5-point reduction in the PCL-M score in, respectively, 47.9% and 31.9% of subjects, and a greater than 10 PCL-M point decline in 33.8% and 27.5% of subjects, respectively (Fig. 2c). In contrast, about 79% of subjects in the present study had greater than a clinically significant 10-point drop in the score (Fig. 2c). The difference between the subjects here and subjects in the previous study [5] was highly significant (p < 0.0001), for both the 5 point and a 10-point PCL score declines. Moreover, the mean 20.6 point 4-week peak change score for all the subjects reported herein substantially exceeded the beneficial outcomes of the VA study which may have been partially attributable to placebo effects [5]. Regardless of the genesis of the 5–10 PCL-M point mean score decrease in the VA study [5], the substantially greater magnitude of the response observed in the current work (24.5 points) renders placebo an unlikely sole cause underlying the therapeutic outcome reported for the present study [5]. Fig. 3 shows anxiety (HAM-A) and depression (HAM-D) scores for those subjects that responded or did not respond to PrTMS in terms of PCL-5 scores. Initially, mean depression and anxiety scores were in the ’severe’ category, and those subjects that showed a PrTMS induced PCL-5 score decline of greater than 5 points with PrTMS had HAM-A and HAM-D scores fall to moderate levels. In the PCL-5 nonresponder group both the HAM-A and the HAM-D scores did not change significantly.

### 3.4 Spectral EEG findings

#### 3.4.1 Alpha band power and center frequency shift

**Fig. 4:**
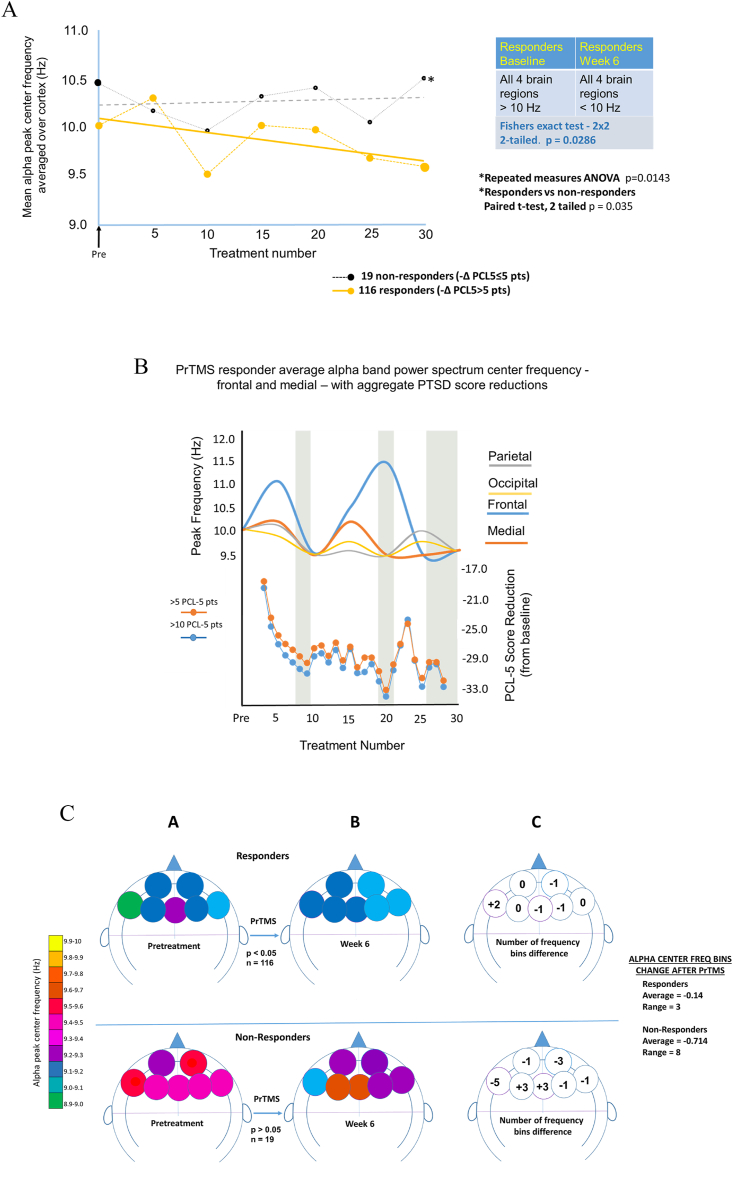
a. PrTMS shift in the dominant alpha peak frequency. Shown according to week of PrTMS treatment for all brain regions for responders (>5 PCL-5 point reduction) and non-responders (≤5 PCl-5 point reduction). The inset indicates that for responders all 4 brain regions, frontal, medial, parietal and occipital, had mean center peak frequencies above 10 Hz while after 6 weeks of PrTMS all regions were below 10 Hz, average = 9.6 Hz. (two-tailed Fishers exact 2 × 2 test, p = 0.0286.) Nonresponders exhibited no significant effect (p = 1.00). A repeated measures ANOVA (over time) indicated that responders and nonresponders differed significantly (p = 0.0143). b. Graph showing alpha center frequency and PCL-5 score versus PrTMS treatment number according to brain cortical region. The low points for the EEG alpha band center frequency appear to generally correspond to the low points of the PCL-5 PTSD self-report questionnaire scores. c. Alpha mean center frequency for each frontal cortical EEG electrode before and after PrTMS. Arbitrarily sized color circles denote alpha center frequency for each EEG lead for 116 responders and 19 nonresponders. Panel A shows the frontal cortical center frequencies before PrTMS, and Panel B indicates center frequencies after treatment. Panel C indicates the frequency bins for each electrode that rose, fell, or remained constant after PrTMS. A nonparametric binomial distribution sign test indicated that responder cortical peak frequencies changed after PrTMS (p = 0.034), while for nonresponders there was no significant difference between before and after treatment (p = 0.153).

The alpha band oscillatory peak center frequency shifted to lower frequencies for the cortex overall in PrTMS responders. In contrast, non-responders did not exhibit an alpha band frequency shift, as illustrated by the frequency versus number of treatment sessions graph in Fig. 4a. Weekly PrTMS peak alpha frequencies differed significantly (repeated measures ANOVA p = 0.0143; paired *t*-test p = 0.035) between responders and nonresponders. This decline in alpha center frequency in responders is consistent with the possibility of a PrTMS associated improvement of DMN connectivity [97].

Fig. 4b depicts the center frequency for each brain region, frontal, medial, parietal, and occipital, along with the change in PCL-5 score for each treatment week according to session, relative to baseline, in responders, over the course of treatment. Initially there is correspondence between the frontal cortex frequency low with the first PCL-5 score nadir, for all subjects, and although the frontal region lags, its alpha center frequency is lowered by 26 treatments as is the PCL-5 score. The PCL-5 score low points for medial, parietal and occipital regions correspond to alpha center frequency lows, and frequency is also reduced by 5–6 weeks. Fig. 4c is a cortical schematic color scale representation of binned alpha band center frequency superimposed on an outline of the cortex, for each frontal EEG lead in PrTMS responders and nonresponders. In Fig. 4c panel A, the average frontal pretreatment variability and center frequency was greater for non-responders. After PrTMS Fig. 4c, **panel B** indicates that for responders the alpha center frequency became more uniform, and showed the expected, normal left right hemispheric difference. The degree of change after PrTMS in terms of frequency bins is shown in Fig. 4c, **panel C** for each EEG electrode. A nonparametric binomial distribution sign test using the binned data indicated that responder peak frequencies (n = 116) differed before and after PrTMS (p = 0.034) but this was not the case for nonresponders (p = 0.153, n = 19).

#### 3.4.2 Alpha band center frequency uniformity

**Fig. 5:**
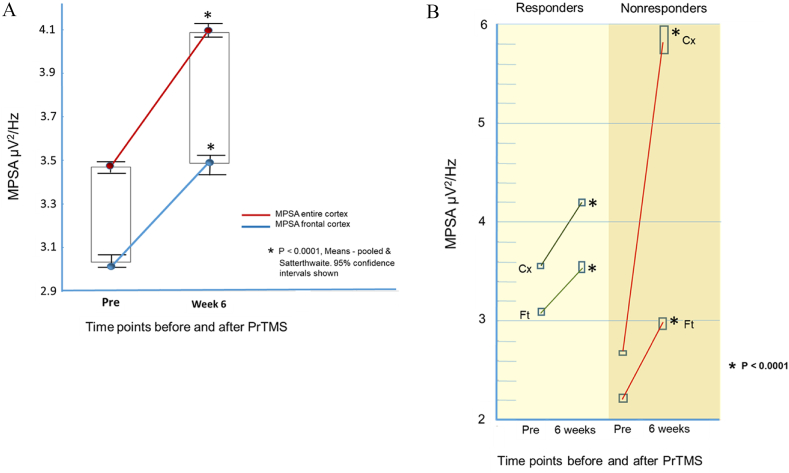
Mean EEG power spectrum amplitudes between 8-13 Hz. Includes all electrodes over the frontal cortex and the entire cortex for both responders (n = 116, 728,688 data points) and nonresponders (n = 19, 101,802 data points). Cx = entire cortex, Ft = frontal cortex. Means for both cortical territories were compared between pretreatment versus 6 weeks, while the week 6 means were compared for responders versus nonresponders. All comparisons were performed via parametric (pooled) and nonparametric (Satterthwaite) tests, because the data were skewed, i.e., not normally distributed. All indicated comparisons revealed significant differences p < 0.0001, and boxes denote 95% confidence intervals.

In responders, the alpha band center frequency declined after PrTMS and was characterized by greater synchrony in the frontal cortex (Fig. 4c). Non-responders had a more irregular center frequency pattern between individual EEG leads, and higher alpha frequencies in the frontal cortex compared to responders, consistent with poorer DMN functioning. Comparison of mean amplitudes between EEG electrodes in the alpha band (8–13 Hz) showed that for the frontal cortex, and the entire brain the mean amplitude was increased and differed significantly before versus after PrTMS (P < 0.0001). This is shown in Fig. 5a for responders (728,688 data points), and **5b** for responders and nonresponders (101,802 data points). After PrTMS nonresponders exhibited lower frontal amplitude than did responders, but for nonresponders the entire cortex apparently was substantially elevated in terms of amplitude, compared to responders (Fig. 5b). It may noteworthy that for nonresponders the differential between the frontal cortex and the whole cortex increased markedly after PrTMS, and if real, this may indicate an impaired connectivity and lack of coordination between the frontal cortical region and the rest of the brain.

#### 3.4.3 Alpha band narrowing

**Fig. 6:**
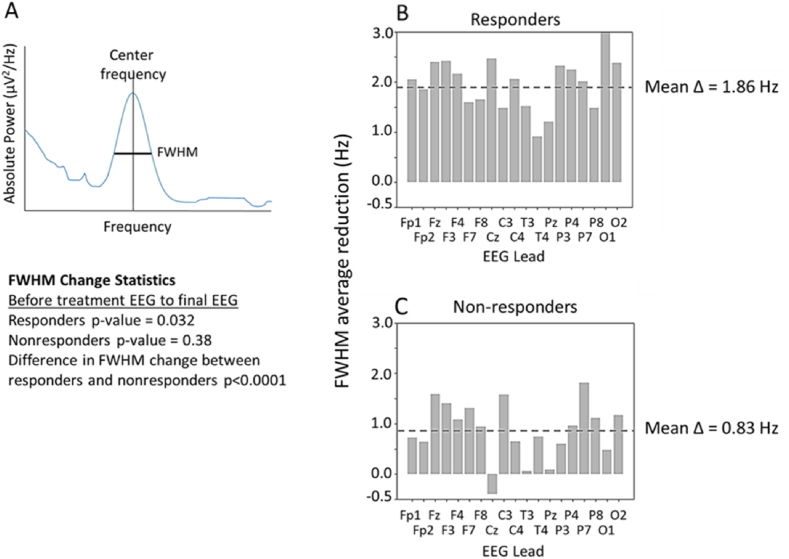
(a) Schematic of FWHM of the dominant alpha peak in the EEG power spectrum. (b) FWHM in Hz averaged for responders. Final PrTMS EEG (Δ PCL5>5), n = 126. (c) FHWM in Hz averaged for non-responders. Final PrTMS EEG (Δ PCL5≤5), n = 44. Statistics shown in figure. Red lines in **b** and **c** denote mean decline for each graph, which differed significantly for responders versus nonresponders (p < 0.0001, repeated measures ANOVA and paired *t*-test).

The alpha oscillatory peak assumes a generally Gaussian shape, and this feature in responders narrowed, or ‘sharpened’, after PrTMS treatment, a commonly observed phenomenon in alpha band neurophysiology [98]. Fig. 6a illustrates schematically how standard full width half-max (FWHM) measurement was applied to the alpha peak in a subset of patients. The reduction in the alpha peak FWHM after PrTMS, for each EEG lead, is depicted in Fig. 6b and c for responders and nonresponders, respectively. This change in responders may reflect increased alpha band firing synchrony of different neuronal clusters. Alternatively, PrTMS may have decreased the level of damping of alpha oscillators, which sharpened the alpha peak [98,99]. Regardless of the exact mechanism, PrTMS could have altered the activity of cortical and subcortical alpha oscillators, and cortical synchrony seemed to have been enhanced. The reduction in FWHM was substantially greater than that observed for non-responders (Repeated measures ANOVA (p < 0.0001) and paired test (p < 0.0001). In future studies it would be interesting to determine whether non-responders differ in terms of drug taking, overeating, gambling, and other addictive behaviors.

**Fig. 7:**
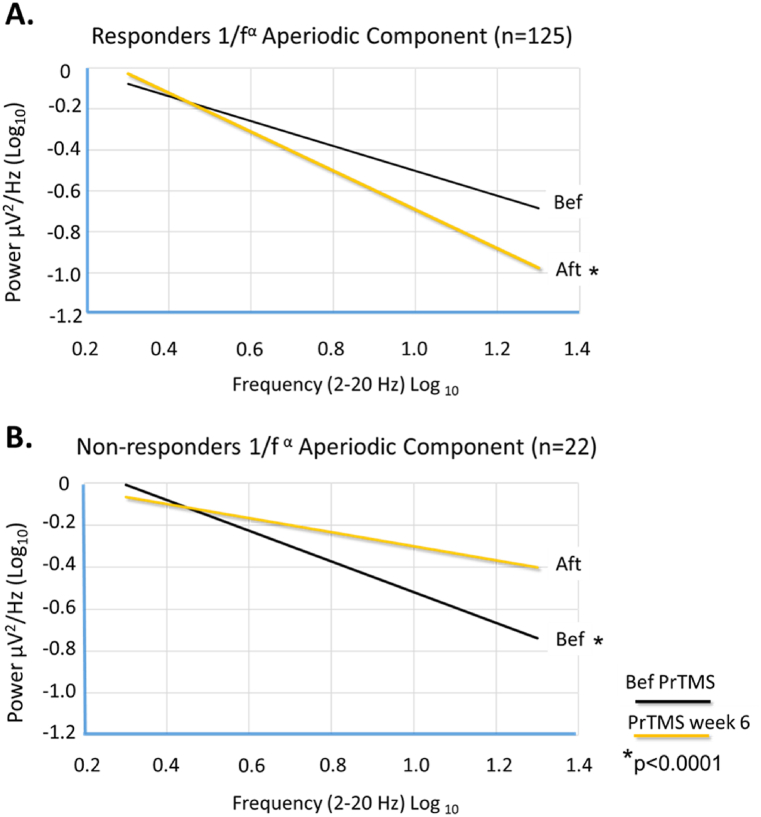
a) Logarithmic robust regressions of the average frontal cortex EEG power spectrum between 2 and 20 Hz, before and after 4 weeks of PrTMS, for 116 patients that were responders, >5 point decline in PLC-5 score. After PrTMS the 1/f aperiodic spectral component clearly declined in responders as the regression line fell more steeply to the right (paired *t*-test pre vs. post p < 0.0001). The robust regression slopes (∝) for responders before and after PrTMS were −0.61 and −0.95, respectively. b) Robust regression for 19 nonresponders. The 1/f aperiodic spectral component clearly increased as the regression line became flatter after PrTMS (paired *t*-test pre vs. post p < 0.0001). The robust regression slopes (∝) for non-responders before and after PrTMS were −0.73 and −0.34, respectively. The steeper regression line after PrTMS in responders suggested that cortical oscillatory synchrony increased and possibly inhibitory GABA activity also increased, while the opposite appeared to have occurred in nonresponders.

The EEG power spectrum 1/f aperiodic component is a measure of the synchronicity of spiking between cortical neuronal populations, it is also reflective of cognitive and perceptual states, excitatory versus inhibitory balance, and it is regarded as a potential biomarker of disease such as attention deficit hyperactivity disorder (ADHD) or schizophrenia [92,95,100]. The aperiodic component was calculated for both PrTMS responders and nonresponders [92]. The aperiodic component for responders is shown in the log-log EEG spectral plot generated by robust regression in Fig. 7a, for the frontal cortex [92,95]. Clearly the aperiodic regression slope became steeper after PrTMS, and aperiodic amplitude, or power, declined. This suggests that cortical neuronal population spiking became more synchronous which is presumed to enhance signaling efficiency, and a shift towards greater inhibition [95,100,101]. To the best of our knowledge, this is the first such demonstration, as the characterization of the 1/f aperiodic component and its treatment related modulation has not been previously reported for PTSD.

In nonresponders the aperiodic component increased during 4 weeks of treatment, as indicated by flattening of the robust regression line in Fig. 7b. The mechanistic substrate for this is not clear but may have involved lowered firing synchrony of neuronal clusters and/or a shift in the relative dominance of specific neurotransmitters and excitatory versus inhibitory signaling pathways. This finding implies that for nonresponders cortical areas may have been activated by rTMS but there was no improvement in terms of coordinated activity and reduced hyper arousal [95]. Moreover, it is interesting that in nonresponders the 1/f slope decreased, i.e., the regression was flatter, indicating comparative excitation, and the cortical power spectrum amplitude shown in Fig. 6b was elevated well above that for responders (p < 0.0001).

## 4 Discussion

This report describes the results obtained in veterans with combat PTSD using a dynamic and personalized form of rTMS that we refer to as PrTMS, based on subject specific continual updating of stimulation frequency, location, and pattern, guided by serial EEG power spectra and neurocognitive exams. This methodology facilitated a substantial decline in PTSD, anxiety, and depression scores, and induced non-subjective EEG spectral effects with potential biomarker and mechanistic significance.

PrTMS was administered because combat PTSD is often resistant to cognitive and pharmacological interventions. The patients were treated whenever they decided to come to the clinic, and very few patients remained after week six, as the dropout rate was high. For the sake of completeness we mentioned these patients. For statistical validity we elected to present and analyze data that had at least ten patients per time point, and such data extended to 27 treatments, almost six weeks, but not beyond. Fig. 2B shows that after 4 weeks of PrTMS there was no further improvement in PCL-5 scores, and the subsequent plateau represents treatment resistant patients. If patients respond they will likely do so during the first few weeks, so in future prospective studies a 4–6 week timeframe should be used. In Fig. 2b the percent not showing improvement after four weeks is about 17% which corresponds almost exactly to the percentage of patients with less than 5% PCL-5 improvement, i.e., no treatment response, with PrTMS. Interestingly, a recent (2022) retrospective literature meta-analysis of rTMS in depression indicated that a 4–6 week treatment duration yields tangible results, and Carpenter and co-workers (2017) reported that significant antidepressant effects were obtained after 4 weeks of rTMS [102,103].

The results acquired here support our primary objectives, which included lowering the mean PTSD PCL-5 score to below the diagnostic threshold of 33, and reducing the spectral EEG alpha band peak center frequency. Secondary endpoints were also attained, including lowering severe depression and anxiety scores to a mild to moderate range, and the identification of spectral EEG correlates of important frontal cortical neurophysiological PrTMS effects e.g., greater EEG alpha band peak amplitude, narrowing of alpha band peak width, and for the first time in PTSD, a steepening of the spectral 1/f^α^ aperiodic component.

The Hollywood blockbuster, “The Hurt Locker,” powerfully highlighted the psychological dilemmas that are so disabling for combat veterans. Despite their chronically fearful states during combat, many veterans upon their return to civilian life despair of never being able to find meaning and perceive contextual subtleties in normal existence against the backdrop of the intense stimulation they felt during combat. These Veterans often see civilian life as bland, stressful, and unfulfilling and describe themselves as “numb.” They may turn to drugs or alcohol as a means of overcoming this stress and numbing and recreating the highs they felt while in combat. Improved DMN connectivity and functionality attained via PrTMS [104] may help patients reprocess traumatic memories and develop new cognitive strategies for reframing and responding to stressful situations [21] and for confronting and recontextualizing traumatic memories and emotions in a safe therapeutic environment [105].

It has previously been suggested that EEG based systems for guiding the placement of transcranial magnetic stimulation (TMS) could lead to more accurate targeting of brain regions, which may improve the effectiveness of TMS for treating neurological and psychiatric conditions [106]. In fact, in a sham-controlled study, EEG analysis identified the optimal target region to guide the placement of the TMS leading to more effective stimulation and a greater reduction in symptoms of major depressive disorder (MDD), which is a common co-morbidity in combat veterans with PTSD [64,107]. Moreover, resting EEG measures were used to differentiate between rTMS responders and non-responders, highlighting the heuristic value of the EEG for predicting rTMS outcomes [108]. In a similar fashion, the EEG has been reported to differentiate rTMS responders and non-responders among patients with severe brain damage that are unable to show meaningful or consistent behavioral responses to their environment [109]. This further suggests that EEG after-treatment effects may likewise be a useful tool for predicting rTMS treatment response [110]. The present study further substantiates the potential value of the EEG in guiding and predicting rTMS efficacy, and extends previously reported finding by suggesting that the beneficial effect may generalize to trauma and stressor-related neuropsychiatric disorders e.g., PTSD.

Significant rTMS-induced improvement in PCL-5 symptomatology is in accordance with an earlier study applying theta burst rTMS [111] and demonstrating EEG based discrimination of active versus sham-treated patients [112]. Although there were methodological similarities between the latter [111] and the current study, e.g., enrollment of veterans and rTMS combined with EEG measures, there were also important differences, including the rTMS protocol, application to combat veterans, and the focus on EEG-guided placement of the magnetic coil. Thus, our independent replication supports the potential of EEG-guided rTMS as an efficacious therapy for PTSD.

Much remains to be learned about the pathophysiology of PTSD, and treatment resistance surely results from a poorly understood and complex natural history characterized by progressively dysregulated interactions between multiple brain signaling hubs. Importantly however, there is emerging evidence that PTSD involves dysrhythmia of the DMN elements in the alpha oscillatory band [113]. This has prompted increasing speculation that PTSD may be initiated and sustained by a loss of brain oscillatory synchronicity and connectivity that in health is supported by the alpha rhythm [26,[114], [115], [116]]. In the foregoing context, rTMS could prove useful partly because it is likely orthogonal to current therapies, and because it may engage a basal cause of PTSD by resetting an ensemble of disrupted thalamocortical alpha frequency generators to stabilize the DMN [60,61,114,117]. Entrainment of thalamocortical oscillators is held to be a major effect of rTMS, and several reports have addressed the relationship between alpha frequency and rTMS [[118], [119], [120]]. The premise for a therapeutic effect created by rTMS may be that alpha band cortical oscillations facilitate coordination between discrete, distributed brain areas at rest and during task coping activities and there is an optimal frequency that is modulated by various factors and brain states [26,61,84] that may be altered in neuropsychiatric disorders such as PTSD.

Our PrTMS methodology aligns with observations made by others, albeit in a modified way, in two key respects: (1) Stimulation of a comparatively greater cortical area to enhance synchronicity [84] by incorporating frequency-specific stimulation of multiple discrete motor sensory and prefrontal cortical sites and (2) Adjustments of cortical stimulus locations and stimulation frequency, on a weekly basis, as therapy progressed. We followed this paradigm and reduced TMS power to avoid overstimulation, which conceptually aligned with the recent report [85] that rTMS could synchronize the cortical alpha frequency band at 80% of resting motor threshold (MT), which is roughly half the lowest conventional rTMS field strength [84,85]. Here we safely expanded the number of pre-frontal, frontal, and motor sensory cortical regions that were stimulated, by reducing machine power to 20–60%.

Continuous updating of stimulation frequency throughout treatment aligns with the view [121] that the alpha peak frequency has multiple cortical sources. From this perspective, the dominant alpha frequency may be a dynamic, changing set-point driven by time varying neurophysiology, rather than remaining static and being fixed solely by thalamic inputs to the cortex [121]. The growing body of evidence certainly adds credence to regulation of alpha frequency by the cortical systems and that state dependent changes may occur via external triggering, such as with rTMS, with important functional and therapeutic ramifications [122].

Hence, with PrTMS, it is the cortical response to successive lower amplitude stimulation, both in terms of objective alpha waveform data and serial neurocognitive exams that dictates further stimulation frequency selection. The frequency target moves over the course of the patient's treatment and thus requires continuous EEG power spectrum monitoring. This procedure in our patients was associated with a decline in the alpha peak center frequency, greater cortical synchrony, a narrower alpha band peak (FWHM), increased alpha amplitude in the frontal cortex, and a reduced 1/f aperiodic alpha band spectral component. These neurophysiological correlates may align with increased synchrony of neuronal population spiking, and less damping of alpha oscillators, and may have resulted in the amplitude increase caused by PrTMS. Importantly, for PTSD the 1/f aperiodic component has not been previously characterized and its reduction observed here may reflect beneficial changes in homeostatic brain signaling pathways [95].

A limitation of the study is the open label design. The placebo effect with TMS stems from patients' expectations and the attention they receive during treatment. The direction of bias is typically positive, with patients experiencing improved neuropsychiatric symptoms. A further constraint is that the comparison to our team's previous VA standard rTMS study is limited, although that initial work was a systematic foray into the use of rTMS methodology for PTSD in combat veterans. Moreover, combat veterans completed self-report questionnaires for PTSD and depression, a reasonable approach, but we did not conduct formal interviews and diagnostic procedures. We also note that the magnitude of the PTSD score reductions we obtained are typically much greater that those observed in the placebo arm of previously reported studies, we obtained non-subjective spectral EEG changes in a relatively sizable group of participants, and we identified clearly contrasting PrTMS responders and nonresponders. The data presented here do show that the primary aims of this work were realized; (1) that PrTMS is safe, and (2) that further prospective studies of PrTMS are warranted and our findings should be brought to the attention of relevant clinical and research communities. Nevertheless, the results reported here should be deemed preliminary pending replication in future prospective sham-controlled studies.

In conclusion, this manuscript describes positive outcomes in combat PTSD achieved via PrTMS treatment. This demonstrates the utility of spectral EEG analysis for the identification of specific brain systems such as the DMN that are affected, and therefore require restorative stimulation. Personalized medicine involves the optimal matching of proper tests to clinically eligible patients, and the exposition of individual EEG rTMS responses may be an important intermediary allowing for a more targeted and personalized treatment. Moreover, EEG-guided PrTMS may allow for the use of lower stimulation intensities and shorter treatment durations, which could further reduce the already low risk of seizures and other adverse effects associated with rTMS. While daily PrTMS treatments for 6 weeks or more represents a time burden, we believe that the potential to safely overcome treatment resistant PTSD justifies the time investment. Overall, our preliminary data suggests that EEG-guided PrTMS holds promise for individualized medicine as it has the potential to optimize treatment efficacy, reduce the risk of adverse effects, and improve patient outcomes for PTSD and possibly for multiple neuropsychiatric disorders.

## Data availability statement

The authors confirm that raw data associated with Fig. 1, Fig. 2, Fig. 3, Fig. 4, Fig. 5, Fig. 6, Fig. 7 inclusive will be made available to interested parties. The raw data incorporated into these figures is entirely anonymous and cannot in any way be linked to identifiable patients. The authors cannot provide individual patient information in the context of the raw data without adequate justification and unless specific HIPAA conforming data access and protection steps are taken by qualified personnel. Moreover, upon request, PeakLogic can provide copies of IRB approvals indicating that PrTMS poses a nonsignificant risk and may be used for research purposes.

## Code availability

The computer code that extracted FWHM information from subject power spectra is available by contacting Dr. S Abbasi (Abbasi@up.edu) at the Department of Electrical Engineering at the University of Portland. Those interested in the code for EEG spectral analyses need to contact Mr. Chad Nybo at CrossTx (Chad@crosstx.com) and Dr. Lori Christman at StatKing Clinical Services (Lori@statkingclinical.com).

## Author contribution statement

Milan Makale; Kenneth Blum; Mark S Gold; David Baron; Igor Elman; Catherine A Dennen: Analyzed and interpreted the data; Wrote the paper. Shaghayegh Abbasi: Analyzed and interpreted the data. Chad Nybo: Contributed reagents, materials, analysis tools or data; Analyzed and interpreted the data. Jason Keifer: Contributed reagents, materials, analysis tools or data. Lori Christman; Jean Lud Cadet: Analyzed and interpreted the data. Kaci Fairchild, Jerome Yesavage: Conceived and designed the experiments. Kevin Murphy: Conceived and designed the experiments; Performed the experiments; Contributed reagents, materials, analysis tools or data.

## Additional information

Supplementary content related to this article has been published online at [URL].

## Declaration of competing interest

The authors declare the following financial interests/personal relationships which may be considered as potential competing interests:Dr. Kevin Murphy owns shares in PeakLogic Inc.

Dr. Milan Makale receives salary compensation from PeakLogic.

Dr. Lori Christman receives salary compensation from StatKing Clinical Services.

Mr. Chad Nybo has shares in, is a founder/owner of, and receives salary from CrossTx.

Dr. Jason Keifer is the owner/operator of BrainHealth Hawaii Inc.

Dr. Kenneth Blum is Executive Chairman of TranspliceGen Therapeutics Inc., a company that has been licensed to develop Blum's entire patent portfolio include genetic testing and pro-dopamine regulation.

Drs Shaghayegh Abbasi, J. Kaci Fairchild, Jerome Yesavage, Mark S. Gold, David Baron, Jean Lud Cadet, Igor Elman, and Catherine A. Dennen do not have any competing interests to disclose. The authors’ immediate family members do not have management/advisory or consulting relationships that in any way relate to this study.
