# Supplementary material for: Personalized repetitive transcranial magnetic stimulation (prtms®) for post-traumatic stress disorder (ptsd) in military combat veterans
Source: Heliyon. 2023 Aug 8;9(8):e18943. doi: 10.1016/j.heliyon.2023.e18943 (PMC10440537; doi:10.1016/j.heliyon.2023.e18943)
Supplement: Multimedia component 1 [file mmc1.pdf]

HAMILTON ANXIETY SCALE (HAM-A)

Patient Name ______________________________ Today's Date ______________

The Hamilton Anxiety Scale (HAM-A) is a rating scale developed to quantify the severity of anxiety symptomatology, often used in psychotropic drug evaluation. It consists of 14 items, each defined by a series of symptoms. Each item is rated on a 5-point scale, ranging from 0 (not present) to 4 (severe).

0 = Not present to 4 = Severe

Score

☐ 1. ANXIOUS MOOD
- Worries
- Anticipates worst

☐ 2. TENSION
- Startles
- Cries easily
- Restless
- Trembling

☐ 3. FEARS
- Fear of the dark
- Fear of strangers
- Fear of being alone
- Fear of animal

☐ 4. INSOMNIA
- Difficulty falling asleep or staying asleep
- Difficulty with Nightmares

☐ 5. INTELLECTUAL
- Poor concentration
- Memory Impairment

☐ 6. DEPRESSED MOOD
- Decreased interest in activities
- Anhedoni
- Insomnia

☐ 7. SOMATIC COMPLAINTS: MUSCULAR
- Muscle aches or pains
- Bruxism

☐ 8. SOMATIC COMPLAINTS: SENSORY
- Tinnitus
- Blurred vision

☐ 9. CARDIOVASCULAR SYMPTOMS
- Tachycardia
- Palpitations
- Chest Pain
- Sensation of feeling faint

☐ 10. RESPIRATORY SYMPTOMS
- Chest pressure
- Choking sensation
- Shortness of Breath

☐ 11. GASTROINTESTINAL SYMPTOMS
- Dysphagia
- Nausea or Vomiting
- Constipation
- Weight loss
- Abdominal fullness

☐ 12. GENITOURINARY SYMPTOMS
- Urinary frequency or urgency
- Dysmenorrhea
- Impotence

☐ 13. AUTONOMIC SYMPTOMS
- Dry Mouth
- Flushing
- Pallor
- Sweating

☐ 14. BEHAVIOR AT INTERVIEW
- Fidgets
- Tremor
- Paces
