# Supplementary material for: Personalized repetitive transcranial magnetic stimulation (prtms®) for post-traumatic stress disorder (ptsd) in military combat veterans
Source: Heliyon. 2023 Aug 8;9(8):e18943. doi: 10.1016/j.heliyon.2023.e18943 (PMC10440537; doi:10.1016/j.heliyon.2023.e18943)
Supplement: Multimedia component 2 [file mmc2.pdf]

HAMILTON DEPRESSION RATING SCALE (HAM-D)

(To be administered by a health care professional)

Patient Name ______________________________ Today's Date ______________

The HAM-D is designed to rate the severity of depression in patients. Although it contains 21 areas, calculate the patient's score on the first 17 answers.

☐ 1. DEPRESSED MOOD
(Gloomy attitude, pessimism about the future, feeling of sadness, tendency to weep)
0 = Absent
1 = Sadness, etc.
2 = Occasional weeping
3 = Frequent weeping
4 = Extreme symptoms

☐ 2. FEELINGS OF GUILT
0 = Absent
1 = Self-reproach, feels he/she has let people down
2 = Ideas of guilt
3 = Present illness is a punishment; delusions of guilt
4 = Hallucinations of guilt

☐ 3. SUICIDE
0 = Absent
1 = Feels life is not worth living
2 = Wishes he/she were dead
3 = Suicidal ideas or gestures
4 = Attempts at suicide

☐ 4. INSOMNIA - Initial
(Difficulty in falling asleep)
0 = Absent
1 = Occasional
2 = Frequent

☐ 5. INSOMNIA - Middle
(Complains of being restless and disturbed during the night. Waking during the night.)
0 = Absent
1 = Occasional
2 = Frequent

☐ 6. INSOMNIA - Delayed
(Waking in early hours of the morning and unable to fall asleep again)
0 = Absent
1 = Occasional
2 = Frequent

☐ 7. WORK AND INTERESTS
0 = No difficulty
1 = Feelings of incapacity, listlessness, indecision and vacillation
2 = Loss of interest in hobbies, decreased social activities
3 = Productivity decreased
4 = Unable to work. Stopped working because of present illness only. (Absence from work after treatment or recovery may rate a lower score).

☐ 8. RETARDATION
(Slowness of thought, speech, and activity; apathy; stupor.)
0 = Absent
1 = Slight retardation at interview
2 = Obvious retardation at interview
3 = Interview difficult
4 = Complete stupor

☐ 9. AGITATION
(Restlessness associated with anxiety.)
0 = Absent
1 = Occasional
2 = Frequent

☐ 10. ANXIETY - PSYCHIC
0 = No difficulty
1 = Tension and irritability
2 = Worrying about minor matters
3 = Apprehensive attitude
4 = Fears

HAMILTON DEPRESSION RATING SCALE (HAM-D)

(To be administered by a health care professional)

☐ **11. ANXIETY - SOMATIC**

Gastrointestinal, indigestion
Cardiovascular, palpitation, Headaches
Respiratory, Genito-urinary, etc.

0 = Absent
1 = Mild
2 = Moderate
3 = Severe
4 = Incapacitating

☐ **12. SOMATIC SYMPTOMS - GASTROINTESTINAL**

(Loss of appetite , heavy feeling in abdomen; constipation)

0 = Absent
1 = Mild
2 = Severe

☐ **13. SOMATIC SYMPTOMS - GENERAL**

(Heaviness in limbs, back or head; diffuse backache; loss of energy and fatiguability)

0 = Absent
1 = Mild
2 = Severe

☐ **14. GENITAL SYMPTOMS**

(Loss of libido, menstrual disturbances)

0 = Absent
1 = Mild
2 = Severe

☐ **15. HYPOCHONDRIASIS**

0 = Not present
1 = Self-absorption (bodily)
2 = Preoccupation with health
3 = Querulous attitude
4 = Hypochondriacal delusions

☐ **16. WEIGHT LOSS**

0 = No weight loss
1 = Slight
2 = Obvious or severe

☐ **17. INSIGHT**

(Insight must be interpreted in terms of patient's understanding and background.)

0 = No loss
1 = Partial or doubtfull loss
2 = Loss of insight

TOTAL ITEMS 1 TO 17: ______________

0 - 7 = Normal
8 - 13 = Mild Depression
14-18 = Moderate Depression
19 - 22 = Severe Depression
$\geq$ 23 = Very Severe Depression

☐ **18. DIURNAL VARIATION**

(Symptoms worse in morning or evening. Note which it is.)

0 = No variation
1 = Mild variation; AM (   ) PM (   )
2 = Severe variation; AM (   ) PM (   )

☐ **19. DEPERSONALIZATION AND DEREALIZATION**

(feelings of unreality, nihilistic ideas)

0 = Absent
1 = Mild
2 = Moderate
3 = Severe
4 = Incapacitating

☐ **20. PARANOID SYMPTOMS**

(Not with a depressive quality)

0 = None
1 = Suspicious
2 = Ideas of reference
3 = Delusions of reference and persecution
4 = Hallucinations, persecutory

☐ **21. OBSESSIONAL SYMPTOMS**

(Obsessive thoughts and compulsions against which the patient struggles)

0 = Absent
1 = Mild
2 = Severe

* Adapted from Hamilton, M. *Journal of Neurology, Neurosurgery, and Psychiatry*. 23:56-62, 1960.
