# Supplementary material for: Personalized repetitive transcranial magnetic stimulation (prtms®) for post-traumatic stress disorder (ptsd) in military combat veterans
Source: Heliyon. 2023 Aug 8;9(8):e18943. doi: 10.1016/j.heliyon.2023.e18943 (PMC10440537; doi:10.1016/j.heliyon.2023.e18943)
Supplement: Multimedia component 3 [file mmc3.pdf]

National Center for PTSD
POSTTRAUMATIC STRESS DISORDER

The PTSD Checklist for *DSM-5* with Criterion A

Version date: 14 August 2013

Reference: Weathers, F. W., Litz, B. T., Keane, T. M., Palmieri, P. A., Marx, B. P., & Schnurr, P. P. (2013). *The PTSD Checklist for DSM-5 (PCL-5) – Extended Criterion A* [Measurement instrument]. Available from http://www.ptsd.va.gov/

URL: http://www.ptsd.va.gov/professional/assessment/adult-sr/ptsd-checklist.asp

This page intentionally left blank

PCL-5 with Criterion A

Instructions: This questionnaire asks about problems you may have had after a very stressful experience involving actual or threatened death, serious injury, or sexual violence. It could be something that happened to you directly, something you witnessed, or something you learned happened to a close family member or close friend. Some examples are a serious accident; fire; disaster such as a hurricane, tornado, or earthquake; physical or sexual attack or abuse; war; homicide; or suicide.

First, please answer a few questions about your worst event, which for this questionnaire means the event that currently bothers you the most. This could be one of the examples above or some other very stressful experience. Also, it could be a single event (for example, a car crash) or multiple similar events (for example, multiple stressful events in a war-zone or repeated sexual abuse).

Briefly identify the worst event (if you feel comfortable doing so):

____

____

____

____

How long ago did it happen? ______________________________ (please estimate if you are not sure)

Did it involve actual or threatened death, serious injury, or sexual violence?

______ Yes

______ No

How did you experience it?

______ It happened to me directly

______ I witnessed it

______ I learned about it happening to a close family member or close friend

______ I was repeatedly exposed to details about it as part of my job (for example, paramedic, police, military, or other first responder)

______ Other, please describe ____

If the event involved the death of a close family member or close friend, was it due to some kind of accident or violence, or was it due to natural causes?

______ Accident or violence

______ Natural causes

______ Not applicable (the event did not involve the death of a close family member or close friend)

Second, below is a list of problems that people sometimes have in response to a very stressful experience. Keeping your worst event in mind, please read each problem carefully and then circle one of the numbers to the right to indicate how much you have been bothered by that problem in the past month.

**In the past month, how much were you bothered by:**	**Not at all**	**A little bit**	**Moderately**	**Quite a bit**	**Extremely**
1. Repeated, disturbing, and unwanted memories of the stressful experience?	0	1	2	3	4
2. Repeated, disturbing dreams of the stressful experience?	0	1	2	3	4
3. Suddenly feeling or acting as if the stressful experience were actually happening again (as if you were actually back there reliving it)?	0	1	2	3	4
4. Feeling very upset when something reminded you of the stressful experience?	0	1	2	3	4
5. Having strong physical reactions when something reminded you of the stressful experience (for example, heart pounding, trouble breathing, sweating)?	0	1	2	3	4
6. Avoiding memories, thoughts, or feelings related to the stressful experience?	0	1	2	3	4
7. Avoiding external reminders of the stressful experience (for example, people, places, conversations, activities, objects, or situations)?	0	1	2	3	4
8. Trouble remembering important parts of the stressful experience?	0	1	2	3	4
9. Having strong negative beliefs about yourself, other people, or the world (for example, having thoughts such as: I am bad, there is something seriously wrong with me, no one can be trusted, the world is completely dangerous)?	0	1	2	3	4
10. Blaming yourself or someone else for the stressful experience or what happened after it?	0	1	2	3	4
11. Having strong negative feelings such as fear, horror, anger, guilt, or shame?	0	1	2	3	4
12. Loss of interest in activities that you used to enjoy?	0	1	2	3	4
13. Feeling distant or cut off from other people?	0	1	2	3	4
14. Trouble experiencing positive feelings (for example, being unable to feel happiness or have loving feelings for people close to you)?	0	1	2	3	4
15. Irritable behavior, angry outbursts, or acting aggressively?	0	1	2	3	4
16. Taking too many risks or doing things that could cause you harm?	0	1	2	3	4
17. Being "superalert" or watchful or on guard?	0	1	2	3	4
18. Feeling jumpy or easily startled?	0	1	2	3	4
19. Having difficulty concentrating?	0	1	2	3	4
20. Trouble falling or staying asleep?	0	1	2	3	4
